# Identification and characterization of a novel chromosome-encoded aminoglycoside *O*-nucleotidyltransferase gene, *ant(9)-Id,* in *Providencia* sp. TYF-12 isolated from the marine fish intestine

**DOI:** 10.3389/fmicb.2024.1475172

**Published:** 2024-12-12

**Authors:** Yan Yu, Runzhi Zhang, Wei Pan, Xinyi Sheng, Susu Chen, Junjun Wang, Junwan Lu, Qiyu Bao, Yunliang Hu, Pengfei Jiang, Dawei Huang

**Affiliations:** ^1^Institute of Biomedical Informatics/School of Laboratory Medicine and Life Sciences, Wenzhou Medical University, Wenzhou, China; ^2^Institute of Molecular Virology and Immunology, Department of Microbiology and Immunology, School of Basic Medical Sciences, Wenzhou Medical University, Wenzhou, China; ^3^Department of Laboratory Sciences, The People’s Hospital of Yuhuan, Yuhuan, China; ^4^Department of Laboratory Sciences, Pingyang Hospital of Wenzhou Medical University, Pingyang, China; ^5^Medical Molecular Biology Laboratory, School of Medicine, Jinhua University of Vocational Technology, Jinhua, China

**Keywords:** *Providencia*, resistance gene, *ant(9)-Id*, aminoglycoside *O*-nucleotidyltransferase, kinetic parameter

## Abstract

**Background:**

The mechanisms underlying the resistance of the genus *Providencia* to aminoglycosides are complex, which poses a challenge for the efficient treatment of infectious diseases caused by these pathogens. To help clinicians treat infections more effectively, a more comprehensive understanding of antibiotic resistance mechanisms is urgently needed.

**Methods:**

Plates were streaked to isolate bacteria from the intestinal contents of fish. The standard agar dilution method was used to determine the minimum inhibitory concentrations (MICs) of the antimicrobial agents. Molecular cloning was carried out to study the function of the novel antibiotic inactivation gene *ant(9)-Id.* The kinetic parameters of ANT(9)*-*Id were measured by a SpectraMax multifunctional microplate reader. Whole-genome sequencing and bioinformatic analysis were conducted to elucidate the sequence structure and evolutionary relationships of similar genes.

**Results:**

The novel aminoglycoside *O*-nucleotidyltransferase gene *ant(9)-Id* was encoded on the chromosome of a species-unclassified isolate designated *Providencia* sp. TYF-12, which was isolated from the intestine of a marine fish. Among the 11 aminoglycosides tested, *ant(9)-Id* was resistant to only spectinomycin. The MIC of spectinomycin for the recombinant strain carrying *ant(9)-Id* (pUCP20-*ant(9)-Id*/DH5α) increased 64-fold compared with that of the control strain (pUCP20/DH5ɑ). ANT(9)*-*Id shares the highest amino acid (aa) identity of 46.70% with the known drug resistance enzyme ANT(9)-Ic. Consistent with the MIC results, ANT(9)*-*Id showed high affinity and catalytic efficiency for spectinomycin, with a *K*_m_ of 8.94 ± 2.50 μM and a *k*_cat_/*K*_m_ of 26.15 μM^−1^·s^−1^. This novel resistance gene and its close homologs are conserved in *Providencia* strains from various sources, including some of clinical significance. No mobile genetic elements (MGEs) surrounding the *ant(9)-Id*(−like) genes were identified.

**Conclusion:**

This work revealed and characterized a novel spectinomycin resistance gene, *ant(9)-Id*, along with its biological features. Identifying novel resistance genes in pathogens can assist in rational medication use and the identification of additional antimicrobial resistance mechanisms in microbial populations.

## Introduction

*Providencia* is a gram-negative bacillus within the family Enterobacteriaceae. The first described organism of this genus was *Bacillus inconstans* (Ornstein, 1920). During the period from 1943 to 1946, organisms of this genus were isolated from patients suffering from enteric diseases and were called “anaerogenic paracolon 29,911” (Stuart et al., 1943). In 1951, the genus *Providencia* was proposed, and this group of strains was therefore reclassified into the genus *Providencia* (O’Hara et al., 2000; Ovchinnikova et al., 2013). This genus currently contains 11 validly named members, such as *P. rustigianii*, *P. heimbachae*, and *P. rettgeri*, and three nonvalidly named species, namely, *P. wenzhouensis* (Zhou et al., 2021), *P. hangzhouensis* (Dong et al., 2023) and *P. entomophila* (Ksentini et al., 2019). These strains reside in polluted water reservoirs, wastewater and soil; they can also be isolated from living organisms and cause animal and human infections, and they can cause severe dyspnea and hemorrhagic pneumonia in piglets (Wang et al., 2014). These strains are also related to cholecystitis and hepatitis in domestic shorthair cats (Newton and Fry, 2018). As conditional pathogens, these strains can cause serious injections in humans, including meningitis and urinary tract infections (UTIs) (Sipahi et al., 2010). *Procidencia* spp. are the third most common cause of elderly urinary tract infections, next to *Escherichia coli* and *Klebsiella* spp. Globally (Vecchi et al., 2013). Among the *Providencia* genus, *P. stuartii*, *P. hangzhouensis* and *P. rettgeri* are significantly correlated with human infections (Dong et al., 2024).

Aminoglycoside antibiotics are broad-spectrum drugs that are mainly employed to treat suspected or confirmed acute serious infections (Serio et al., 2018). These antimicrobial agents exert bactericidal effects by attaching to the 16S rRNA within the target prokaryotic cell to halt the synthesis of bacterial proteins (Aradi et al., 2020; Doi et al., 2016). Resistance to aminoglycosides has become increasingly severe due to the abuse of antibiotics. The mechanisms underlying resistance to aminoglycosides include mainly aminoglycoside-modifying enzymes (AMEs) (Ramirez and Tolmasky, 2010), decreased antibiotic permeability (Donkor et al., 2023), increased endogenous drug efflux (Morita et al., 2012) and 16S rRNA methyltransferase activity (Kawai et al., 2021; Srinivas et al., 2023). Enzymatic modification is one of the most popular mechanisms leading to drug inactivation (Zárate et al., 2018). AMEs are divided into three families according to their modification positions: aminoglycoside *N*-acetyltransferases (AACs), aminoglycoside *O*-nucleotidyltransferases (ANTs), and aminoglycoside *O*-phosphotransferases (APHs). ANTs mediate drug inactivation by a nucleotide to a hydroxyl of the aminoglycoside (Ramirez and Tolmasky, 2010). Currently, there are five major categories of ANTs, including ANT(6), ANT(9), ANT(4′), ANT(3″) and ANT(2″), which catalyze hydroxyl group adenylation at corresponding positions. Because some Enterobacteriaceae, such as *Providencia* spp. *Morganella morganii,* can express high levels of AmpC beta-lactamases, which confer resistance to penicillin-like and cephalosporin antibiotics, the use of penicillins and cephalosporins should be avoided, and alternative therapy with aminoglycosides, quinolones or carbapenems is recommended (Harris and Ferguson, 2012; Liu et al., 2020).

In this study, through genomic sequencing and molecular cloning, an uncharacterized aminoglycoside *O*-nucleotidyltransferase gene, named *ant(9)-Id*, which was discovered in a *Providencia* isolate isolated from a marine fish intestine sample, was identified. The enzyme kinetic parameters of ANT(9)-Id were also studied.

## Materials and methods

### Bacterial strains and plasmids

In recent years, to investigate bacterial resistance to antimicrobials, more than 300 isolates have been isolated from marine samples in Wenzhou, Zhejiang Province, China. One isolate, designated *Providencia* sp. TYF-12, was obtained from a fish intestine. 16S RNA gene homology comparison, whole-genome average nucleotide identity (ANI) and digital DNA–DNA hybridization (dDDH) analyses were used for the identification of the bacteria. The strains and plasmids used in this study are listed in Table 1.

**Table 1:** Bacteria and plasmids used in this work.

Strain or plasmid	Relevant characteristic	Reference
TYF-12	The wild-type *Providencia* sp. TYF-12	This study
*E. coli* DH5ɑ (DH5ɑ)	A host for cloning the *ant(9)-Id* gene	Our laboratory collection
*E. coli* BL21 (BL21)	A host for expressing Ant(9)-Id	Our laboratory collection
*E. coli* ATCC 25922	A quality control for antimicrobial susceptibility test	Our laboratory collection
pUCP20-*ant(9)-Id*/DH5α	DH5α carrying the recombinant plasmid pUCP20-*ant(9)-Id*	This study
pCold I-*ant(9)-Id*/BL21	BL21 carrying the recombinant plasmid pCold I-*ant(9)-Id*	This study
pUCP20	Cloning vector for the PCR products of the *ant(9)-Id* gene with its upstream promoter region, AMP^r^	Our laboratory collection
pCold I	Expression vector for the PCR products of the ORF of the *ant(9)-Id* gene, AMP^r^	Our laboratory collection

r, resistance; AMP, ampicillin; ORF, open reading frame.

### Antimicrobial susceptibility testing

In accordance with the standards of the Clinical and Laboratory Standards Institute (CLSI),^1^ the agar dilution method was used to determine the minimum inhibitory concentration (MIC). *E. coli* ATCC 25922 served as a quality control for antibiotic susceptibility testing. The tested antimicrobial agents included spectinomycin, tobramycin, streptomycin, netilmicin, paromomycin and amikacin (Table 2).

**Table 2:** MICs of 17 antimicrobials for 5 strains (μg/mL).

Drug class	Antimicrobial	ATCC25922	DH5ɑ	pUCP20/DH5ɑ	pUCP20-*ant(9)-Id*/DH5α	*Providencia* sp.TYF-12
Aminoglycoside	Spectinomycin	8	8	8	512	>1,024
	Gentamicin	0.5	0.5	0.5	0.06	2
	Tobramycin	1	0.5	0.5	0.06	2
	Streptomycin	4	4	4	2	32
	Kanamycin	2	2	2	2	256
	Paromomycin	4	4	4	0.5	128
	Neomycin	2	2	1	0.5	8
	Sisomicin	≤1	≤1	≤1	≤1	≤1
	Amikacin	≤2	≤2	≤2	≤2	≤2
	Netilmicin	0.5	0.5	0.5	0.25	8
	Ribostamycin	4	≤2	≤2	≤2	256
β-Lactam	Cefazolin	2	1	1	/	≤0.25
	Cefoxitin	4	4	4	/	1
	Ceftriaxone	0.06	0.03	0.03	/	0.125
	Cefepime	0.06	0.03	0.03	/	0.015
	Imipenem	0.125	0.125	0.125	/	1
	Aztreonam	0.25	0.06	0.06	/	≤0.015

### Sequencing and annotation of the genome sequences

The genomic DNA of *Providencia* sp. TYF-12 was sequenced on the Illumina HiSeq 2,500 and PacBio RS II platforms by Shanghai Personal Biotechnology Co., Ltd. (Shanghai, China). The long PacBio reads were first assembled via Unicycler (Wick et al., 2017) and then corrected with Illumina sequencing data via pilon (Walker et al., 2014). Prokka v1.14.6 (Seemann, 2014) was used for the prediction of the open reading frames (ORFs). Potential proteins were annotated via the NCBI nonredundant protein database and DIAMOND v2.0.11 (Buchfink et al., 2021). Antimicrobial resistance genes were predicted via Resistance Gene identifier v5.2.0^2^ and the comprehensive antibiotic resistance database (CARD) (McArthur et al., 2013). The ANI was calculated via fastANI v1.33 (Jain et al., 2018). The genetic environment of *ant(9)-Id* and its homologous genes were analyzed via clinker v0.0.24 (Gilchrist and Chooi, 2021). The multiple sequence alignment diagram and phylogenetic tree were generated via MAFFT v7.490 and MEGAX (Katoh and Standley, 2013; Kumar et al., 2018), respectively. The molecular weight and pI of ANT(9)-Id were predicted via the JavaScript program (Stothard, 2000).

### Molecular cloning of the resistance gene

The target gene, along with its promoter region, was amplified via PCR with the primers listed in Table 3. The PCR products were subsequently inserted into the pUCP20 vector via T4 DNA ligase (Takara Bio, Inc., Dalian, China). The recombinant plasmid was introduced into *E. coli* DH5α cells via the calcium transformation method, and the transformant were subsequently grown on Luria–Bertani (LB) solid media supplemented with ampicillin (AMP) at a final concentration of 100 μg/mL. The inserted sequence was confirmed via first-generation sequencing (Shanghai Sunny Biotechnology Co., Ltd., Shanghai, China).

**Table 3:** Primers for cloning the *ant(9)-Id* gene.

Primer^a^	Sequence (5′-3′)^b^	Restriction endonuclease	Vector	Annealing temperature (°C)	Amplicon size (bp)
pro-*ant(9)-Id*-F	TATTCTATTCAGGGTTTATGGAGCGCAG		pUCP20	61	1,240
pro-*ant(9)-Id*-R	ACAAATTCTATCTGTTGAAACAAATAACGC		pUCP20		1,240
orf-*ant(9)-Id*-F	CGCGGATCCGACGACGACGACAAGATGAAAAATTCATATCAGGTAGCG	*Bam*HI + EK enzyme	pCold I	55	868
orf-*ant(9)-Id*-R	CCCAAGCTTATCTAAGTATCAGACAAATTCTATCTGTTG	*Hind*III	pCold I		868

^a^Primers with “pro” were used to clone the *ant(9)-Id* gene with its promoter region. Primers with “orf” were used to clone the ORF of the *ant(9)-Id* gene. ^b^The underlined sequences represent the restriction sites and their protective bases.

### Expression and purification of the recombinant protein ANT(9)-Id

To obtain the protein ANT(9)-Id, PCR was used for amplification of the ORF of the *ant(9)-Id* gene. The amplified product and the expression vector pCold I were both digested by the restriction enzymes *Bam*HI and *Hind*III, and they were ligated. The recombinant plasmid (pCold I-*ant(9)-Id*) was transformed into *E. coli* BL21 cells. The transformants were screened on LB agar plates containing 100 μg/mL AMP, and the cloned fragment was verified by first-generation sequencing. The recombinant strain (*E. coli* BL21/pCold I-*ant(9)-Id*) was cultured overnight in LB broth supplemented with 100 μg/mL AMP at 37°C and then continuously cultured in LB liquid medium at a ratio of 1:100 until the optical density (OD_600_) reached 0.6 to 0.8. Sterile isopropyl-beta-D-thiogalactopyranoside (IPTG; Sigma Chemicals Co., St. Louis, MO, United States) was added to a final concentration of 0.5 mM, and protein expression was induced in a shaker incubator at 16°C. The bacteria were collected by centrifugation (8,000 × g, 10 min) and then lysed by ultrasonication for 2 min, after which the supernatant was collected. The target protein in the supernatant was purified with a His-tag protein purification kit (Beyotime, Shanghai, China). At 25°C, the samples were treated with recombinant enterokinase (EK enzyme) for 2 h, after which the His-tag was removed. The purity and relative molecular mass of the enzyme were evaluated by sodium dodecyl sulfate–polyacrylamide gel electrophoresis (SDS–PAGE). The protein concentration of ANT(9)-Id was determined via a BCA protein assay kit (Beyotime, Shanghai, China).

### Kinetic studies of the enzyme ANT(9)-Id

The catalytic activity of ANT(9)-Id was analyzed by coupling the enzyme to UDP-glucose pyrophosphorylase (UGP), phosphoglucomutase (PGM) and glucose-6-phosphate dehydrogenase (G6PD). The increase in NADPH was measured at a wavelength of 340 nm via a SpectraMax multifunctional microplate reader (M5, Molecular Devices, America) to monitor the progress of the reaction. The total reaction system was 0.2 mL and contained a mixture of enzymes and substrates, including 2 U/mL UGP, 20 U/mL PGM, 20 U/mL G6PD, 10 mM MgCl_2_, 50 mM HEPES (pH 7.5), 500 μM dithiothreitol (DTT), 500 μM UDP-glucose, 500 μM glucose 1,6-bisphosphate, 500 μM NADP, 2 mM ATP and purified ANT(9)-Id protein. The mixture was incubated at 37°C for 5 min, and a series of concentrations of spectinomycin were finally added to initiate the reaction. The enzyme kinetic parameters (*k*_cat_ and *K*_m_) were determined by fitting the Michaelis–Menten equation via Prism (v8.0.2) software (Chen et al., 2019).

## Results and discussion

### Identification of potential novel aminoglycoside resistance genes

The MICs of the 11 antibiotics for the isolates were tested. Approximately 140 isolates with different resistance spectra were randomly selected (Supplementary Table S1), and the genome sequence of each isolate was sequenced on the Illumina HiSeq 2,500 platform. The potential resistance genes in these genomes were predicted (Supplementary Table S2). In this study, our attention was focused on uncovering aminoglycoside resistance mechanisms. According to the annotated antimicrobial resistance gene profiles derived from the genomic sequences of the 140 isolates, 11 prospective aminoglycoside resistance-related genes with <80% aa identity to the functionally characterized resistance genes in the CARD were selected, which included *aadA9*-, *ant(9)-Ia-*, *aadA25*-, *aph(3′)-Ia-, aac(3)-Id-, aac(6′)-IIa-, aac(6′)-Ic-, aac(3)-Ia-, aac(3)-IV-, aac(2′)-Ia-,* and *aac(6′)-If-*homologous genes. These genes were subsequently cloned, and their resistance phenotypes were tested. *In vitro* antimicrobial susceptibility testing revealed that the *ant(9)-Ia-*like gene (designated *ant(9)-Id* in this work) was functional.

### *ant(9)-Id* confers resistance to spectinomycin

The novel resistance gene *ant(9)-Id* consists of 765 bp and encodes a protein of 254 aa (Supplementary Figure S1), which has a molecular weight of 28.67 kDa and an isoelectric point of 5.03. The results of the antimicrobial susceptibility test conducted on the recombinant strain (pUCP20-*ant(9)-Id*/DH5α) indicated that, of the 11 aminoglycosides tested, the strain demonstrated resistance to spectinomycin only. Compared with that of the control strain (pUCP20/DH5α), the MIC of spectinomycin (512 μg/mL) for the recombinant strain increased 64-fold. As we know that the vector pUCP20 is commonly used to analyze the function of a gene. As it is a high copy number plasmid, the *in vivo* protein concentration encoded by the cloned resistance gene (*ant(9)-Id*) in the recombinant (pUCP20-*ant(9)-Id*/DH5α) would be higher than that of the original host TYF-12 where the resistance gene is located in the chromosome which indicates that one cell has only one copy of the resistance gene. As a result, the MIC level increase of the recombinant carrying the cloned resistance gene (pUCP20-*ant(9)-Id*/DH5α) to an antimicrobial would be higher than that of the original host. The expression vector pCold I, however, was used to express the protein, so the MIC level of the recombinant (pCold I-*ant(9)-Id*/BL21) to the antimicrobial was not tested. As expected, the *in vitro* catalytic activity of ANT(9)-Id aligned with the *in vivo* MIC data of the *ant(9)-Id* gene. The enzyme selectively adenylates spectinomycin, with a *K*_m_ of 8.94 ± 2.50 μM and *k*_cat_/*K*_m_ of 26.15 μM^−1^·s^−1^. However, no adenosine transfer effect on spectinomycin or tobramycin was observed (Table 4 and Supplementary Table S3).

**Table 4:** Kinetic parameters of ANT(9)-Id.

Substrate	*k*_cat_ (s^−1^)	*K*_m_ (μM)	k_cat_/*K*_m_ (μM^−1^·s^−1^)
Spectinomycin	231.57 ± 59.80	8.94 ± 2.50	26.15 ± 2.95
Streptomycin	NH*	NH*	NH*
Tobramycin	NH*	NH*	NH*

*NH, no detectable hydrolysis.

The resistance characteristics of *ant(9)-Id* were consistent with those of the other *ant(9)* genes. At present, four *ant(9)* genes, namely, *ant(9)-Ia* (formerly named *aad9* or *spw*)*, ant(9)-Ib*, *ant(9)-Ic* and *spd,* are available in the CARD database. All of these strains were resistant to streptomycin only (Mahbub Alam et al., 2005; Murphy, 1985; LeBlanc et al., 1991; Sheng et al., 2023; Jamrozy et al., 2014; Wendlandt et al., 2013). Moreover, the proteins ANT(9)-Ib and ANT(9)-Ic also showed specific adenosine transfer effects on spectinomycin (Sheng et al., 2023).

The drug resistance phenotypes of the ANT family are diverse. ANT(9)s mediate resistance to spectinomycin alone (Mahbub Alam et al., 2005). ANT(6)s are resistant to streptomycin (Heo et al., 2022). ANT(3″)s confer resistance to both streptomycin and spectinomycin (Kehrenberg et al., 2005), whereas ANT(2″)s and ANT(4′)s adenylate multiple antimicrobial agents. ANT(2″)s are resistant to gentamicin, tobramycin, kanamycin and other aminoglycoside antibiotics (Wright and Serpersu, 2006); however, the presence of ANT(4′)s enables the host to be resistant to isepamicin, amikacin, tobramycin and other aminoglycoside antibiotics (Jacoby et al., 1990). AACs (Mayer, 1986) and APHs (Ramirez and Tolmasky, 2010) also have a wide substrate spectrum, except APH(9)s, which, like ANT(9)s, confer resistance to spectinomycin only (Fong et al., 2010).

### Genome features, species classification and resistance characteristics of the isolate TYF-12

To investigate the molecular characteristics of the *ant(9)-Id* gene coding sequence, the entire genome of the isolate TYF-12, which carried the novel resistance gene, was sequenced. Free of a plasmid, the whole genome of TYF-12 contains a chromosome that is 4,660,160 bp in length, which encodes 4,613 coding sequences (CDSs) with an average GC content of 46.13% (Table 5).

**Table 5:** General features of the *Providencia* sp. TYF-12 genome.

Description	Chromosome
Size (bp)	4,660,160
GC content (%)	46.13
Predicted coding sequences (CDSs)	4,402
Known proteins	2,975
Hypothetical proteins	1,427
Protein coding (%)	94.46
Average ORF length (bp)	891
Average protein length (aa)	300
tRNAs	154
rRNA operons	(16S-23S-5S) × 43

The 16S rRNA gene sequence of TYF-12 shared the closest relationship, with 99.47% identity and 98.0% coverage, with that of *Providencia vermicola* OP1 (NR_042415.1), followed by those of *P. rettgeri* DSM4541 (NR_042413.1, 99.27% identity and 96.0% coverage) and *P. rettgeri* NCTC11801 (NR_115880.1, 99.19% identity and 96.0% coverage). However, ANI analysis revealed that the genome of TYF-12 shared the greatest identity of 83.99% with that of *P. rettgeri* NCTC 11801 (GCF_900455085.1, not a type strain), followed by 81.6% with that of the type strain *P. vermicola* DSM 17385 (GCF_020381325.1). The dDDH analysis results were consistent with the ANI results, with the highest similarities of 53.3% with *P. rettgeri* NCTC11801 and 49.1% with *P. vermicola* DSM 17385. None of the genome sequences in the public database reached the threshold of ANI (96.0%) (Ciufo et al., 2018) or dDDH (70%) (Moore et al., 1987) for the classification of TYF-12 into an existing species. This finding indicated that this isolate represents a new species within the genus *Providencia* and was thus temporarily designated *Providencia* sp. TYF-12.

In addition to the novel resistance gene *ant(9)-Id* identified in this work, which encodes a protein with the highest aa similarity of 40.91% (87.6% coverage and 46.7% identity) with ANT(9)-Ic (QWQ57435.1) among the functionally characterized proteins, a total of 9 antimicrobial resistance genes with ≥80% nucleotide similarity to the functionally characterized genes found in the CARD database were identified from the whole genome, including two lincosamide genes (*inuF* and *inuG*), five aminoglycoside genes (*aadA*, *aac(6′)-Ib-cr6, aph(3′)-Ia, aph(4)-Ia* and *aac(3)-IVa*), one rifamycin (*arr-3*) and one macrolide (*mphE*) resistance gene (Supplementary Table S4). *In vitro* susceptibility analysis revealed that *Providencia* sp. TYF-12 was resistant to 5 (spectinomycin, streptomycin, kanamycin, paromomycin and ribostamycin) of the 17 antimicrobial agents tested. The aminoglycoside resistance phenotype was consistent with the genotype of the isolate (Supplementary Table S5).

### Phylogenetic relationships and molecular characteristics of the ANT proteins

**Figure 1:**
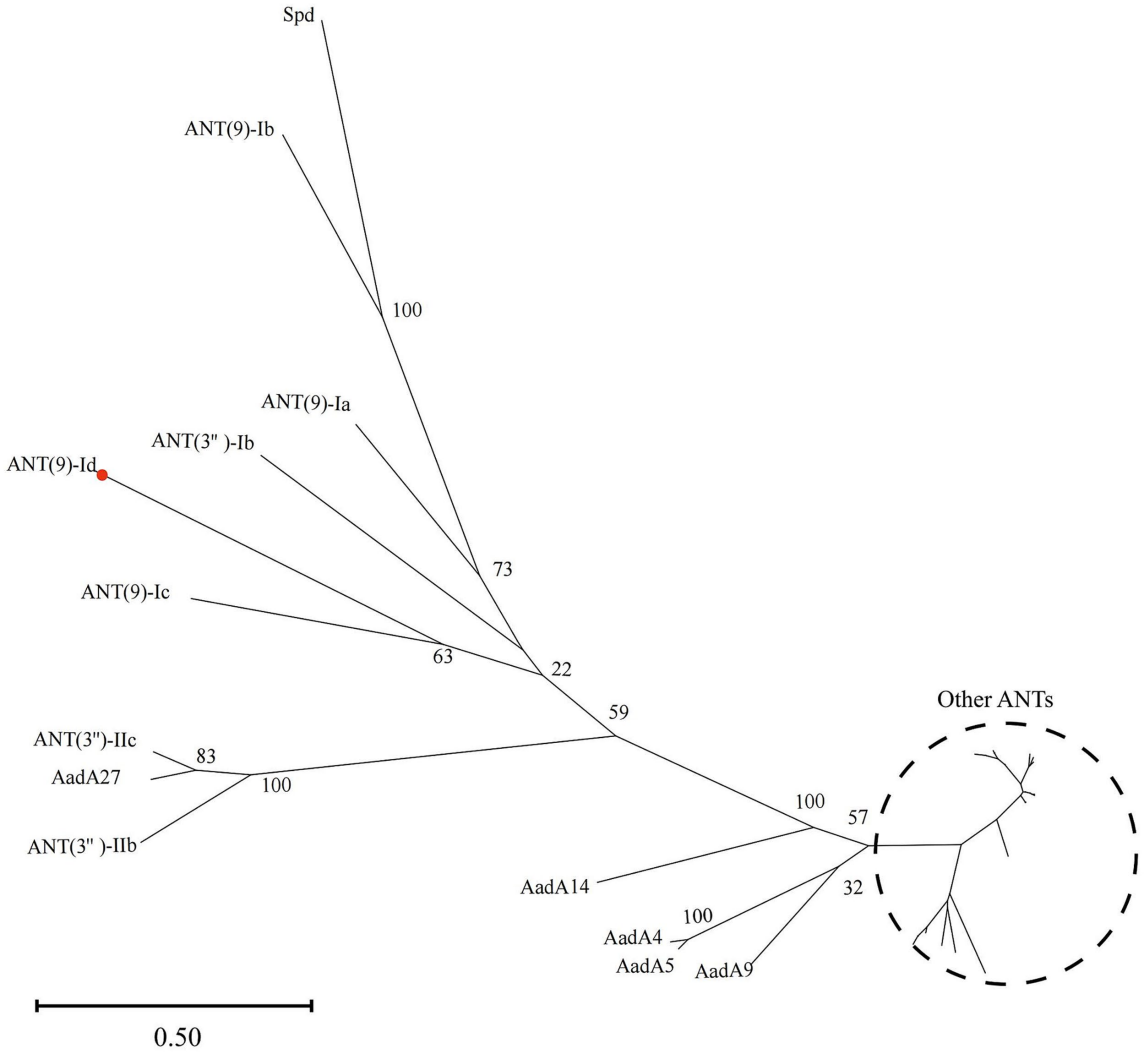
Phylogenetic tree showing the relationships of ANT(9)-Id with other functionally characterized ANTs. ANT(9)-Id is highlighted with a red dot. Other ANTs include AadA (AAO49597.1), AadA2 (AAF27727.1), AadA3 (AAC14728.1), AadA4 (AAN34365.1), AadA5 (AAF17880.1), AadA6 (CAJ32504.1), AadA6/AadA10 (CAJ32491.1), AadA7 (BAD00739.1), AadA8 (AAN41439.1), AadA8b (CAJ13568.1), AadA9 (ABG49324.1), AadA10 (AAL36430.1), AadA11 (AAV32840.1), AadA12 (ACJ47200.1), AadA13 (ABW91178.1), AadA14 (CAI57696.1), AadA15 (ABD58917.1), AadA16 (ACF17980.1), AadA17 (ACK43806.1), AadA21 (AAN87151.1), AadA22 (CAK12750.1), AadA23 (CAH10847.1), AadA24 (ABG72894.1), AadA25 (AET15272.1), AadA27 (CTQ57092.1), ANT(3″)-Ib (QEQ43477.1), ANT(3″)-IIa (CAA26199.1), ANT(3″)-IIc (ENU37733.1), ANT(3″)-IIb (ENU91137.1), ANT(9)-Ia (CAA26428.1), ANT(9)-Ib (AAA16527.1), ANT(9)-Ic (QWQ57435.1), and Spd (AGW81558.1).

A phylogenetic tree of ANT(9)-Id with 34 other ANTs (containing 5 ANT(9)s and 29 ANT(3)s proteins with aa identity >30% with ANT(9)-Id) was reconstructed. At present, a total of 46 functionally characterized ANT family proteins are present in the CARD. They consisted of 2 ANT(2)s (4.35%, 2/46), 31 ANT(3)s (67.39%, 31/46), 4 ANT(4)s (8.70%, 4/46), 5 ANT(6)s (10.87%, 5/46) and 5 ANT(9)s (10.87%, 5/46). Among them, ANT(9)-Ic (QWQ57435.1) shared the highest aa similarity of 41.09% with ANT(9)-Id, followed by ANT(9)-Ia (CAA26428.1, 36.85%) and ANT(3)-Ib (QEQ43477.1, 34.08%), while the other sequences had similarities ranging from 29.64–34.03%. The phylogenetic tree shows that ANT(9)-Id has a close phylogenetic relationship with ANT(9)-Ia and ANT(9)-Ic; this further confirmed that the newly discovered resistance gene was indeed a member of the ANT(9) family (Figure 1).

**Figure 2:**
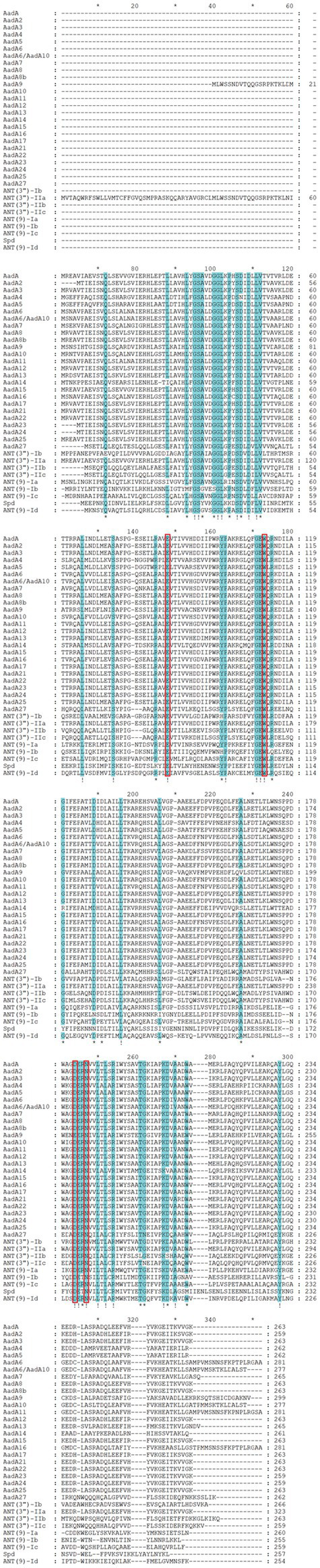
Multiple sequence alignment of ANT(9)-Id with other relatives. Exclamations indicate fully conserved residues. Asterisks indicate strongly similar residues. Gaps are represented via hyphens. The numbers on the right represent the corresponding sequence length. The red frames indicate functional residues. The proteins are as follows: AadA, AadA2, AadA3, AadA4, AadA5, AadA6, AadA6/AadA10, AadA7, AadA8, AadA8b, AadA9, AadA10, AadA11, AadA12, AadA13, AadA14, AadA15, AadA16, AadA17, AadA21, AadA22, AadA23, AadA24, AadA25, AadA27, ANT(3″)-Ib, ANT(3″)-IIa, ANT(3″)-IIc, ANT(3″)-IIb, ANT(9)-Ia, ANT(9)-Ib, ANT(9)-Ic, and Spd. The accession numbers of the proteins are the same as those in Figure 1.

Multiple sequence alignment of ANT(9)-Id with its close relatives revealed that the functionally essential residues of the ANT(9) proteins were conserved in ANT(9)-Id. In AadA (Q8ZPX9), the amino acid residues W173 and D178 are highly important in the adenylation of streptomycin. In the case of spectinomycin, the essential amino acid residues E87, W112, D182 and either H185 or N185 play vital roles (Stern et al., 2018). The last four residues are conserved in ANT(9) family enzymes, including ANT(9)-Id (E82, W107, D174 and N177) (Figure 2).

### Distribution of *ant(9)-Id*-like genes

**Figure 3:**
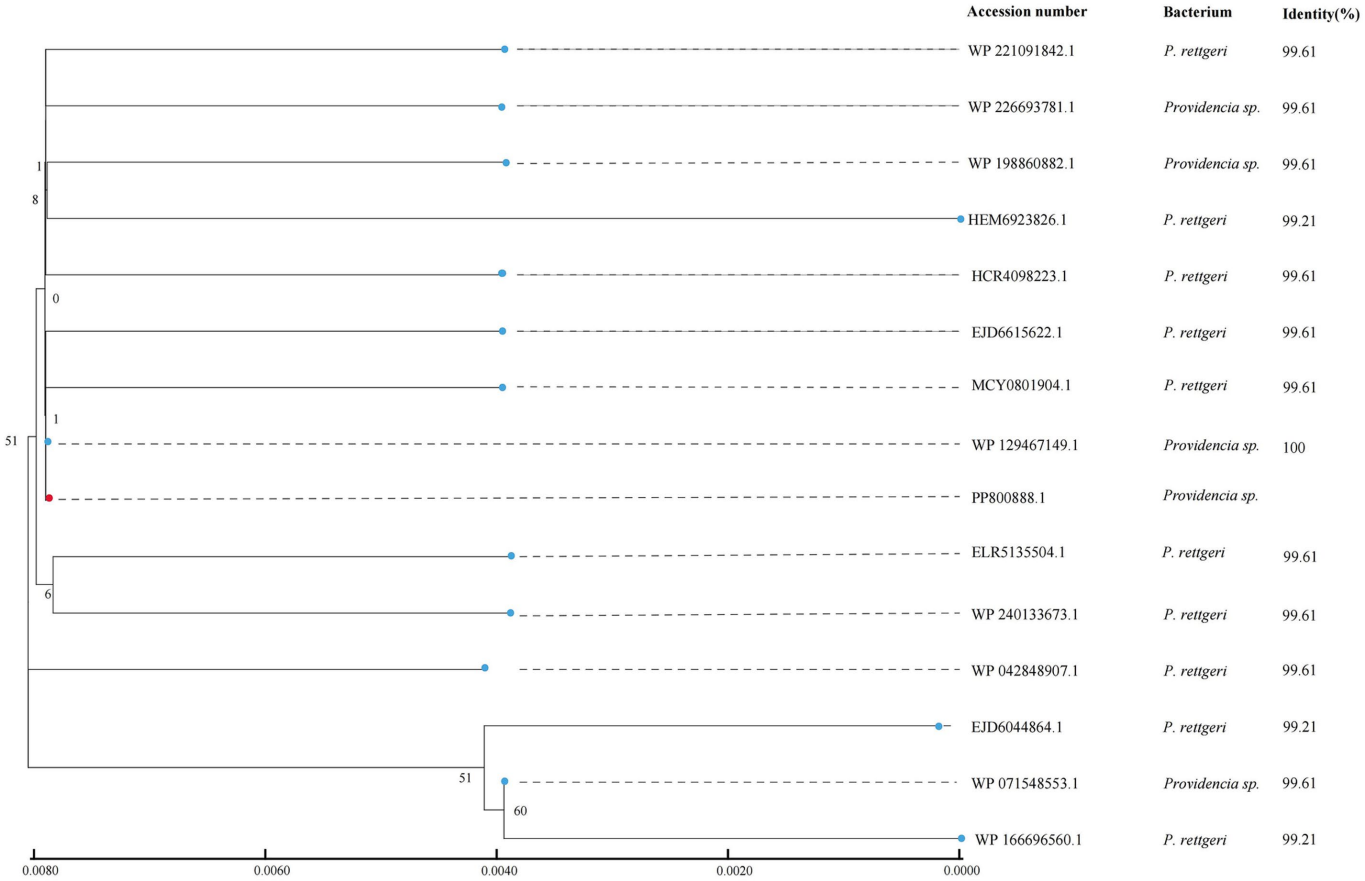
Phylogenetic tree showing the relationships of ANT(9)-Id with other potential ANTs. ANT(9)-Id is highlighted with a red dot.

To analyze the distribution of the *ant(9)-Id*-like genes, the ANT(9)-Id amino acid sequence was used as a query to search for homologous proteins in the NCBI nonredundant protein database. A total of 14 hits that shared ≥99.21% similarity with ANT(9)-Id were identified, and all of them were derived from the genus *Providencia* (Supplementary Table S6). The amino acid sequence of the ANT(9)-Id protein was most similar (100.0%) to that of an aminoglycoside adenylyltransferase family protein (WP_129467149.1), whereas for the remaining 13 proteins, 10 and 3 shared 99.61 and 99.21% similarity, respectively (Figure 3). No protein sharing a similarity ranging from 99.21 to 70.0% was present. These 15 *ant(9)-Id*(−like) genes (including one from this work) were from bacteria of different sources. They were obtained mainly from human clinical samples (60.0%, 9/15). One of these samples was from marine fish (6.7%, 1/15); however, the sources of the other 5 samples were unknown (33.3%, 5/15) (Supplementary Table S6). Further analysis demonstrated that there were about 89 WP_129467149.1 proteins from the different *Providencia* species present in the database,^2^ which included *P. rettgeri*, *P. huashanensis*, *P. xianensis*, *P. alcalifaciens* and several species-undefined strains. Different from the one of this work from the marine fish, the others were isolated from human clinical specimens, soil and so on (Supplementary Table S7).

### Analysis of the genetic context of the *ant(9)-Id*(−like) genes

**Figure 4:**
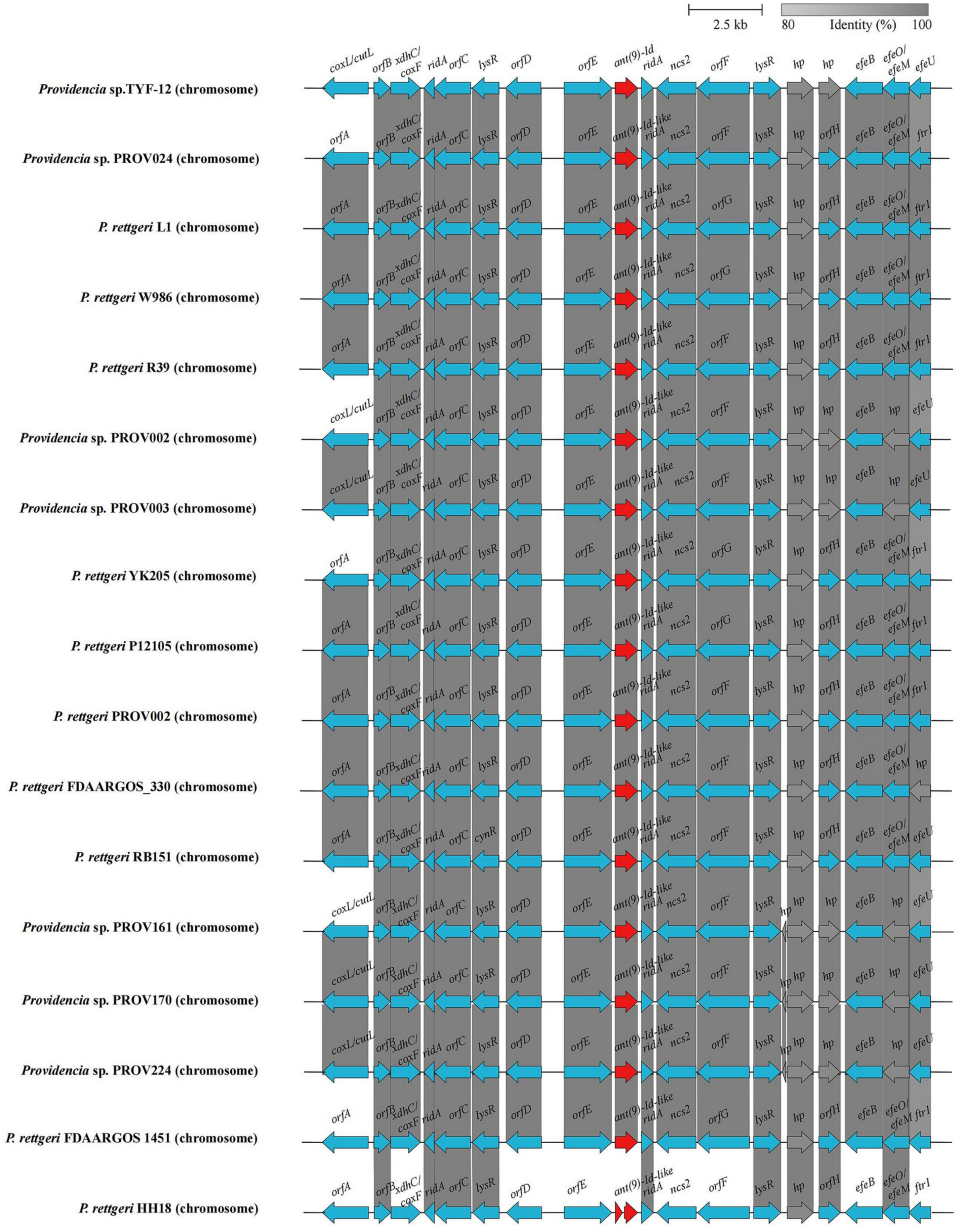
Genetic environment of the *ant(9)-Id*(−like) genes. Regions with ≥80.0% amino acid identity are colored gray. The *ant(9)-Id*(−like) genes are colored red. The accession numbers of the sequence sources are as follows: *Providencia* sp. PROV002 (CP096371.1), *Providencia* sp. PROV003 (CP096369.1), *Providencia* sp. PROV024 (CP120545.1), *Providencia* sp. PROV161 (CP096318.1), *Providencia* sp. PROV170 (CP096313.1), *P. rettgeri* 12,105 (CP109846.1), *P. rettgeri* FDAARGOS 1451 (CP077388.1), *P. rettgeri* PROV002 (CP059345.1), *P. rettgeri* L1 (CP087584.1), *P. rettgeri* YK205 (CP090217.1), *P. rettgeri* R39 (CP066315.1), *P. rettgeri* W986 (CP076258.1), *P. rettgeri* RB151 (CP017671.1), *P. rettgeri* FDAARGOS_330 (CP027418.1), and *P. rettgeri* HH18 (CP054158.1). *hp*, hypothetical protein; *orfA*, molybdopterin-dependent oxidoreductase; *orfB*, nucleotidyltransferase family protein; *orfC*, D-amino acid dehydrogenase; *orfD*, sodium/glutamate symporter; *orfE*, benzoylformate decarboxylase; *orfF*, adenine deaminase; *orfG*, amidohydrolase family protein; *orfH*, VWA domain-containing protein.

To analyze the genetic background of the *ant(9)-Id*(−like) genes, the sequence approximately 20 kb in length with the *ant(9)-Id* gene at the center was intercepted and used as a query to search for similar sequences in the NCBI nucleotide database. Sixteen hits with similarities ranging from 99.37 to 99.72% (> 99.0% identity and 100.0% coverage) were retrieved, each of which encoded an *ant(9)-Id*(−like) gene. Among the 16 sequences, 62.5% (10/16) were from *Providencia rettgeri,* and the rest (37.5%, 6/16) were from species-unclassified *Providencia* strains (Supplementary Table S8). Except for these 16 sequences, no sequences shared ≥80% identity, and ≥ 80% coverage was found. Further structural analysis revealed that these fragments encoded genes related to the following functional categories: transcriptional regulator (*lysR* family), RidA/YER057c/UK114 superfamily protein, ferrous iron transport permease and so on. No mobile genetic element (MGE) was found within the fragments. These sequences exhibited high consistency in both gene content and gene order, which revealed that the *ant(9)-Id*(−like) gene-encoding sequences are highly conserved in the bacteria of the genus *Providencia* (Figure 4).

## Conclusion

During this study, a novel aminoglycoside *O*-nucleotidyltransferase gene named *ant(9)-Id* was identified from the species-unclassified *Providencia* isolate TYF-12. As a gene of the *ant(9)* family, *ant(9)-Id* confers resistance to spectinomycin alone, and it shares the highest aa similarity with *ant(9)-Ic*. Genetic context analysis revealed that the *ant(9)-Id*(−like) genes are not related to a mobile genetic element and are conserved in the microbes of the genus *Providencia,* especially the species *P. rettgeri.* These findings indicate that this phenomenon might contribute to the intrinsic resistance mechanisms of the bacteria of *Providencia.* The discovery of a novel aminoglycoside *O*-nucleotidyltransferase gene, *ant(9)-Id*, can help us to further understand the distributions of the *ant(9)* family and the mechanisms of resistance in opportunistic clinical pathogens.

## Data Availability

The datasets presented in this study can be found in online repositories. The names of the repository/repositories and accession number(s) can be found in the article/Supplementary material.

## Footnotes

^1^ https://clsi.org

^2^ https://www.ncbi.nlm.nih.gov/protein/WP_129467149.1

## References

[ref1] Aradi K. Di Giorgio A. Duca M. (2020). Aminoglycoside conjugation for RNA targeting: antimicrobials and beyond. Chem A Eur J 26, 12273–12309. doi: 10.1002/chem.202002258, PMID: 32539167

[ref2] Buchfink B. Reuter K. Drost H.-G. (2021). Sensitive protein alignments at tree-of-life scale using DIAMOND. Nat. Methods 18, 366–368. doi: 10.1038/s41592-021-01101-x, PMID: 33828273 PMC8026399

[ref3] Chen Q. Zhou W. Qian C. Shen K. Zhu X. Zhou D.. (2019). OXA-830, a novel chromosomally encoded extended-Spectrum class D β-lactamase in *Aeromonas simiae*. Front. Microbiol. 10:2732. doi: 10.3389/fmicb.2019.02732 31849884 PMC6902050

[ref4] Ciufo S. Kannan S. Sharma S. Badretdin A. Clark K. Turner S.. (2018). Using average nucleotide identity to improve taxonomic assignments in prokaryotic genomes at the NCBI. Int. J. Syst. Evol. Microbiol. 68, 2386–2392. doi: 10.1099/ijsem.0.002809, PMID: 29792589 PMC6978984

[ref5] Doi Y. Wachino J. Arakawa Y. (2016). Aminoglycoside Resistance. Infect. Dis. Clin. N. Am. 30, 523–537. doi: 10.1016/j.idc.2016.02.011, PMID: 27208771 PMC4878400

[ref6] Dong X. Jia H. Yu Y. Xiang Y. Zhang Y. (2024). Genomic revisitation and reclassification of the genus *Providencia*. mSphere 9, e00731–e00723. doi: 10.1128/msphere.00731-23 PMC10964429 38412041

[ref7] Dong X. Yu Y. Liu J. Cao D. Xiang Y. Bi K.. (2023). Whole-genome sequencing provides insights into a novel species: *Providencia hangzhouensis* associated with urinary tract infections. Microbiol Spectr 11, e01227–e01223. doi: 10.1128/spectrum.01227-23 37732781 PMC10581081

[ref8] Donkor G. Y. Anderson G. M. Stadler M. Tawiah P. O. Orellano C. D. Edwards K. A.. (2023). A novel ruthenium-silver based antimicrobial potentiates aminoglycoside activity against *Pseudomonas aeruginosa*. mSphere 8, e00190–e00123. doi: 10.1128/msphere.00190-23 37646510 PMC10597350

[ref9] Fong D. H. Lemke C. T. Hwang J. Xiong B. Berghuis A. M. (2010). Structure of the antibiotic resistance factor Spectinomycin phosphotransferase from *Legionella pneumophila*. J. Biol. Chem. 285, 9545–9555. doi: 10.1074/jbc.M109.038364, PMID: 20089863 PMC2843205

[ref10] Gilchrist C. L. M. Chooi Y.-H. (2021). Clinker & clustermap.js: Automatic generation of gene cluster comparison figures. 10.1093/bioinformatics/btab007 33459763

[ref11] Harris P. N. A. Ferguson J. K. (2012). Antibiotic therapy for inducible AmpC ␤-lactamase-producing gram-negative bacilli: what are the alternatives to carbapenems, quinolones and aminoglycosides? Int. J. Antimicrob. Agents. doi: 10.1016/j.ijantimicag.2012.06.004, PMID: 22824371

[ref12] Heo G. Kong H. Kim N. Lee S. Sul S. Jeong D.-W.. (2022). Antibiotic susceptibility of *Bacillus velezensis*. FEMS Microbiol. Lett. 369:fnac017. doi: 10.1093/femsle/fnac017 35167684

[ref13] Jacoby G. A. Blaser M. J. Santanam P. Hächler H. Kayser F. H. Hare R. S.. (1990). Appearance of amikacin and tobramycin resistance due to 4′-aminoglycoside nucleotidyltransferase [ANT(4′)-II] in gram-negative pathogens. Antimicrob. Agents Chemother. 34, 2381–2386. doi: 10.1128/AAC.34.12.2381, PMID: 1965106 PMC172065

[ref14] Jain C. Rodriguez-R L. M. Phillippy A. M. Konstantinidis K. T. Aluru S. (2018). High throughput ANI analysis of 90K prokaryotic genomes reveals clear species boundaries. Nat. Commun. 9:5114. doi: 10.1038/s41467-018-07641-9 30504855 PMC6269478

[ref15] Jamrozy D. M. Coldham N. G. Butaye P. Fielder M. D. (2014). Identification of a novel plasmid-associated spectinomycin adenyltransferase gene spd in methicillin-resistant *Staphylococcus aureus* ST398 isolated from animal and human sources. J. Antimicrob. Chemother. 69, 1193–1196. doi: 10.1093/jac/dkt510, PMID: 24402501

[ref16] Katoh K. Standley D. M. (2013). MAFFT multiple sequence alignment software version 7: improvements in performance and usability. Mol. Biol. Evol. 30, 772–780. doi: 10.1093/molbev/mst010, PMID: 23329690 PMC3603318

[ref17] Kawai A. Suzuki M. Tsukamoto K. Minato Y. Doi Y. (2021). Functional and structural characterization of acquired 16S rRNA methyltransferase NpmB1 conferring Pan-aminoglycoside resistance. Antimicrob. Agents Chemother. 65, e01009–e01021. doi: 10.1128/AAC.01009-21 34310216 PMC8448102

[ref18] Kehrenberg C. Catry B. Haesebrouck F. De Kruif A. Schwarz S. (2005). Novel Spectinomycin/streptomycin resistance gene, aadA14, from *Pasteurella multocida*. Antimicrob. Agents Chemother. 49, 3046–3049. doi: 10.1128/AAC.49.7.3046-3049.2005, PMID: 15980396 PMC1168649

[ref19] Ksentini I. Gharsallah H. Sahnoun M. Schuster C. Hamli Amri S. Gargouri R.. (2019). *Providencia entomophila* sp. nov., a new bacterial species associated with major olive pests in Tunisia. PLoS One 14:e0223943. doi: 10.1371/journal.pone.0223943, PMID: 31639141 PMC6805009

[ref20] Kumar S. Stecher G. Li M. Knyaz C. Tamura K. (2018). MEGA X: molecular evolutionary genetics analysis across computing platforms. Mol. Biol. Evol. 35, 1547–1549. doi: 10.1093/molbev/msy096, PMID: 29722887 PMC5967553

[ref21] LeBlanc D. J. Lee L. N. Inamine J. M. (1991). Cloning and nucleotide base sequence analysis of a spectinomycin adenyltransferase AAD(9) determinant from *Enterococcus faecalis*. Antimicrob. Agents Chemother. 35, 1804–1810. doi: 10.1128/AAC.35.9.1804, PMID: 1659306 PMC245272

[ref22] Liu J. Wang R. Fang M. (2020). Clinical and drug resistance characteristics of *Providencia stuartii* infections in 76 patients. J. Int. Med. Res. 10.1177/0300060520962296 PMC7588764 33081537

[ref23] Mahbub Alam M. Kobayashi N. Ishino M. Sumi A. Kobayashi K.-I. Uehara N.. (2005). Detection of a novel *aph(2″)* allele (*aph[2″]-Ie*) conferring high-level gentamicin resistance and a Spectinomycin resistance gene ant(9)-Ia (aad9) in clinical isolates of enterococci. Microb. Drug Resist. 11, 239–247. doi: 10.1089/mdr.2005.11.239, PMID: 16201926

[ref24] Mayer K. H. (1986). Review of epidemic aminoglycoside resistance worldwide. Am. J. Med. 80, 56–64. doi: 10.1016/0002-9343(86)90480-8, PMID: 3089005

[ref25] McArthur A. G. Waglechner N. Nizam F. Yan A. Azad M. A. Baylay A. J.. (2013). The comprehensive antibiotic resistance database. Antimicrob. Agents Chemother. 57, 3348–3357. doi: 10.1128/AAC.00419-13, PMID: 23650175 PMC3697360

[ref26] Moore W. E. C. Stackebrandt E. Kandler O. Colwell R. R. Krichevsky M. I. Truper H. G.. (1987). Report of the ad hoc committee on reconciliation of approaches to bacterial systematics. Int. J. Syst. Evol. Microbiol. 37, 463–464. doi: 10.1099/00207713-37-4-463

[ref27] Morita Y. Tomida J. Kawamura Y. (2012). Primary mechanisms mediating aminoglycoside resistance in the multidrug-resistant *Pseudomonas aeruginosa* clinical isolate PA7. Microbiology 158, 1071–1083. doi: 10.1099/mic.0.054320-0, PMID: 22282519

[ref28] Murphy E. (1985). Nucleotide sequence of a spectinomycin adenyltransferase AAD(9) determinant from Staphylococcus aureus and its relationship to AAD(3″) (9). Mol. Gen. Genet. 200, 33–39. doi: 10.1007/BF00383309, PMID: 2993813

[ref29] Newton P. L. Fry D. R. (2018). Successful treatment of *Providencia rettgeri* cholecystitis and neutrophilic cholangitis in a cat. J Feline Med Surg Open Reports 4:205511691775076. doi: 10.1177/2055116917750763 PMC5788108 29399368

[ref30] O’Hara C. M. Brenner F. W. Miller J. M. (2000). Classification, identification, and clinical significance of *Proteus*, *Providencia*, and *Morganella*. Clin. Microbiol. Rev. 13, 534–546. doi: 10.1128/CMR.13.4.534 11023955 PMC88947

[ref9001] Ornstein M. (1920). Zur bakteriologie des schmitzbazillus. Z Hyg. 91, 152–178.

[ref31] Ovchinnikova O. G. Rozalski A. Liu B. Knirel Y. A. (2013). O-antigens of bacteria of the genus *Providencia*: structure, serology, genetics, and biosynthesis. Biochemistry Moscow 78, 798–817. doi: 10.1134/S0006297913070110, PMID: 24010842

[ref32] Ramirez M. S. Tolmasky M. E. (2010). Aminoglycoside modifying enzymes. Drug Resist. Updat. 13, 151–171. doi: 10.1016/j.drup.2010.08.003, PMID: 20833577 PMC2992599

[ref33] Seemann T. (2014). Prokka: rapid prokaryotic genome annotation. Bioinformatics 30, 2068–2069. doi: 10.1093/bioinformatics/btu153, PMID: 24642063

[ref34] Serio A. W. Keepers T. Andrews L. Krause K. M. (2018). Aminoglycoside revival: review of a historically important class of antimicrobials undergoing rejuvenation. EcoSal Plus 8:1. doi: 10.1128/ecosalplus.esp-0002-2018, PMID: 30447062 PMC11575671

[ref35] Sheng X. Lu W. Li A. Lu J. Song C. Xu J.. (2023). ANT(9)-Ic, a novel chromosomally encoded aminoglycoside Nucleotidyltransferase from *Brucella intermedia*. Microbiol Spectr 11, e00620–e00623. doi: 10.1128/spectrum.00620-23 37039640 PMC10269693

[ref36] Sipahi O. R. Bardak-Ozcem S. Ozgiray E. Aydemir S. Yurtseven T. Yamazhan T.. (2010). Meningitis due to *Providencia stuartii*. J. Clin. Microbiol. 48, 4667–4668. doi: 10.1128/JCM.01349-10, PMID: 20980575 PMC3008472

[ref37] Srinivas P. Nosrati M. Zelinskaya N. Dey D. Comstock L. R. Dunham C. M.. (2023). 30S subunit recognition and G1405 modification by the aminoglycoside-resistance 16S ribosomal RNA methyltransferase RmtC. Proc. Natl. Acad. Sci. USA 120:e2304128120. doi: 10.1073/pnas.2304128120, PMID: 37307464 PMC10288597

[ref38] Stern A. L. Van Der Verren S. E. Kanchugal P. S. Näsvall J. Gutiérrez-de-Terán H. Selmer M. (2018). Structural mechanism of AadA, a dual-specificity aminoglycoside adenylyltransferase from *Salmonella enterica*. J. Biol. Chem. 293, 11481–11490. doi: 10.1074/jbc.RA118.003989, PMID: 29871922 PMC6065190

[ref39] Stothard P. (2000). The sequence manipulation suite: Java script programs for analyzing and formatting protein and DNA sequences. Bio Techniques 28, 1102–1104. doi: 10.2144/00286ir01 10868275

[ref40] Stuart C. A. Wheeler K. M. Rustigian R. Zimmerman A. (1943). Biochemical and antigenic relationships of the Paracolon Bacteria. J. Bacteriol. 45, 101–119. doi: 10.1128/jb.45.2.101-119.1943, PMID: 16560614 PMC373720

[ref41] Vecchi E. D. Sitia S. Romano C. L. Ricci C. Mattina R. Drago L. (2013). Aetiology and antibiotic resistance patterns of urinary tract infections in the elderly: a 6-month study. J. Med. Microbiol. 62, 859–863. doi: 10.1099/jmm.0.056945-0 23475904

[ref42] Walker B. J. Abeel T. Shea T. Priest M. Abouelliel A. Sakthikumar S.. (2014). Pilon: an integrated tool for comprehensive microbial variant detection and genome assembly improvement. PLoS One 9:e112963. doi: 10.1371/journal.pone.0112963, PMID: 25409509 PMC4237348

[ref43] Wang X. Wang J. Hao H. Qiu L. Liu H. Chen S.. (2014). Pathogenic *Providencia alcalifaciens* strain that causes fatal hemorrhagic pneumonia in piglets. Curr. Microbiol. 68, 278–284. doi: 10.1007/s00284-013-0470-y, PMID: 24129837

[ref44] Wendlandt S. Li B. Lozano C. Ma Z. Torres C. Schwarz S. (2013). Identification of the novel spectinomycin resistance gene spw in methicillin-resistant and methicillin-susceptible *Staphylococcus aureus* of human and animal origin. J. Antimicrob. Chemother. 68, 1679–1680. doi: 10.1093/jac/dkt081, PMID: 23511231

[ref45] Wick R. R. Judd L. M. Gorrie C. L. Holt K. E. (2017). Unicycler: resolving bacterial genome assemblies from short and long sequencing reads. PLoS Comput. Biol. 13:e1005595. doi: 10.1371/journal.pcbi.1005595, PMID: 28594827 PMC5481147

[ref46] Wright E. Serpersu E. H. (2006). Molecular determinants of affinity for aminoglycoside binding to the aminoglycoside Nucleotidyltransferase (2″)-Ia. Biochemistry 45, 10243–10250. doi: 10.1021/bi060935d, PMID: 16922499

[ref47] Zárate S. De La Cruz Claure M. Benito-Arenas R. Revuelta J. Santana A. Bastida A. (2018). Overcoming aminoglycoside enzymatic resistance: Design of Novel Antibiotics and Inhibitors. Molecules 23:284. doi: 10.3390/molecules23020284, PMID: 29385736 PMC6017855

[ref48] Zhou K. Liang J. Dong X. Zhang P. Feng C. Shi W.. (2021). Identification and characterization of a novel chromosomal aminoglycoside 2′-N-acetyltransferase, AAC(2′)-if, from an isolate of a novel *Providencia* species, *Providencia wenzhouensis* R33. Front. Microbiol. 12:711037. doi: 10.3389/fmicb.2021.711037, PMID: 34867838 PMC8640171

