# Supplementary material for: Identification and characterization of a novel chromosome-encoded aminoglycoside O-nucleotidyltransferase gene, ant(9)-Id, in Providencia sp. TYF-12 isolated from the marine fish intestine
Source: Front Microbiol. 2024 Dec 12;15:1475172. doi: 10.3389/fmicb.2024.1475172 (PMC11669914; doi:10.3389/fmicb.2024.1475172)
Supplement: Supplementary file 7 [file Table_4.docx]

TABLE S4 | Resistance genes annotated in the *Providencia* *sp****.*** TYF-12 genome.

| **Gene Family** | **Gene Name** | **Coverage (%)** | **Identity (%)** | **Similarity (%)** |
| --- | --- | --- | --- | --- |
| Lincosamide nucleotidyltransferase (LNU) | *lnuF* | 100.0 | 99.3 | 99.3 |
| Aminoglycoside *O*-nucleotidyltransferases [ANT(3'')] | *aadA* | 100.0 | 100.0 | 100.0 |
| Aminoglycoside *N*-acetyltransferases [AAC(3)] | *aac(3)-IVa* | 100.0 | 100.0 | 100.0 |
| Aminoglycoside *O*-phosphotransferases [APH(4)] | *aph(4)-Ia* | 100.0 | 100.0 | 100.0 |
| Rifampin ADP-ribosyltransferase (Arr) | *arr-3* | 100.0 | 100.0 | 100.0 |
| AAC(6');AAC(6')-Ib-cr | *aac(6')-Ib-cr6* | 100.0 | 99.0 | 99.0 |
| Aminoglycoside *O*-phosphotransferases (APH(3')) | *aph(3')-Ia* | 100.0 | 98.5 | 98.5 |
| Macrolide phosphotransferase (MPH) | *mphE* | 100.0 | 100.0 | 100.0 |
| Lincosamide nucleotidyltransferase (LNU) | *lnuG* | 97.4 | 100.0 | 97.4 |
