# Supplementary material for: Identification and characterization of a novel chromosome-encoded aminoglycoside O-nucleotidyltransferase gene, ant(9)-Id, in Providencia sp. TYF-12 isolated from the marine fish intestine
Source: Front Microbiol. 2024 Dec 12;15:1475172. doi: 10.3389/fmicb.2024.1475172 (PMC11669914; doi:10.3389/fmicb.2024.1475172)
Supplement: Supplementary file 8 [file Table_5.docx]

TABLE S5 | Genotypes of the aminoglycosides resistance genes predicted in the *Providencia* *sp****.*** TYF-12 genome.

| **Antimicrobials** | *aadA* | *aac(3)-IVa* | *aph(4)-Ia* | *aac(6')-Ib-cr6* | *aph(3')-Ia* |
| --- | --- | --- | --- | --- | --- |
| Spectinomycin | + |  |  |  |  |
| Gentamicin |  | + |  |  |  |
| Tobramycin |  | + |  | + |  |
| Streptomycin | + |  |  |  | + |
| Kanamycin |  | + |  | + |  |
| Paromomycin |  |  |  |  | + |
| Neomycin |  |  |  |  | + |
| Sisomicin |  |  |  |  |  |
| Amikacin |  |  |  | + |  |
| Netilmicin |  | + |  | + |  |
| Ribostamycin |  |  |  |  | + |
| Hygromycin |  |  | + |  |  |

+ Positive drug resistance.
