# Supplementary material for: Identification and characterization of a novel chromosome-encoded aminoglycoside O-nucleotidyltransferase gene, ant(9)-Id, in Providencia sp. TYF-12 isolated from the marine fish intestine
Source: Front Microbiol. 2024 Dec 12;15:1475172. doi: 10.3389/fmicb.2024.1475172 (PMC11669914; doi:10.3389/fmicb.2024.1475172)
Supplement: Supplementary file 9 [file Table_6.docx]

TABLE S6 | Distribution and sources of the *ant(9)-Id*-like genes.

| **Description** | **Accession number** | **Identity (%)** | **Coverage (%)** | **Genus** | **Source** |
| --- | --- | --- | --- | --- | --- |
| Aminoglycoside adenylyltransferase family protein | WP_129467149.1 | 100.0 | 100 | *Providencia rettgeri*/*Providencia* sp | Human/soil/... |
| Aminoglycoside adenylyltransferase family protein | MCY0801904.1 | 99.61 | 100 | *Providencia rettgeri* | Human |
| DUF4111 domain-containing protein | EJD6615622.1 | 99.61 | 100 | *Providencia rettgeri* | Human |
| Aminoglycoside adenylyltransferase family protein | WP_042848907.1 | 99.61 | 100 | *Providencia rettgeri* | Human |
| TPA: DUF4111 domain-containing protein | HCR4098223.1 | 99.61 | 100 | *Providencia rettgeri* | Unknown |
| Aminoglycoside adenylyltransferase family protein | WP_221091842.1 | 99.61 | 100 | *Providencia rettgeri* | Human |
| Aminoglycoside adenylyltransferase family protein | WP_226693781.1 | 99.61 | 100 | *Providencia rettgeri/Providencia* sp | Human |
| Aminoglycoside adenylyltransferase family protein | WP_071548553.1 | 99.61 | 100 | *Providencia rettgeri/Providencia* sp | Human |
| DUF4111 domain-containing protein | ELR5135504.1 | 99.61 | 100 | *Providencia rettgeri* | Human |
| Aminoglycoside adenylyltransferase family protein | WP_198860882.1 | 99.61 | 100 | *Providencia rettgeri/Providencia* sp | Unknown |
| TPA: DUF4111 domain-containing protein | HEM6923826.1 | 99.21 | 100 | *Providencia rettgeri* | Unknown |
| Aminoglycoside adenylyltransferase family protein | WP_240133673.1 | 99.61 | 100 | *Providencia rettgeri* | Human |
| DUF4111 domain-containing protein | EJD6044864.1 | 99.21 | 100 | *Providencia rettgeri* | Human |
| Aminoglycoside adenylyltransferase family protein | WP_166696560.1 | 99.21 | 100 | *Providencia rettgeri* | Unknown |
