# Supplementary material for: Identification and characterization of a novel chromosome-encoded aminoglycoside O-nucleotidyltransferase gene, ant(9)-Id, in Providencia sp. TYF-12 isolated from the marine fish intestine
Source: Front Microbiol. 2024 Dec 12;15:1475172. doi: 10.3389/fmicb.2024.1475172 (PMC11669914; doi:10.3389/fmicb.2024.1475172)
Supplement: Supplementary file 11 [file Table_8.docx]

TABLE S8 | Sources of the sequences for the genetic environment analysis.

| **Sequence** | **Accession number** | **Identity (%)** | **Coverage (%)** | **Source** |
| --- | --- | --- | --- | --- |
| *P.rettgeri* FDAARGOS  1451 chromosome | CP077388.1 | 99.72 | 100 | unknown |
| *P.rettgeri* L1 chromosome | CP087584.1 | 99.66 | 100 | Oil contaminated soil |
| *P.rettgeri* FDAARGOS 330 chromosome | CP027418.1 | 99.66 | 100 | human |
| *Providencia* sp. PROV161 chromosome | CP096318.1 | 99.65 | 100 | human |
| *Providencia* sp. PROV170 chromosome | CP096313.1 | 99.65 | 100 | human |
| *Providencia* sp. PROV224 chromosome | CP096302.1 | 99.65 | 100 | human |
| *P.rettgeri* RB151 chromosome | CP017671.1 | 99.65 | 100 | human |
| *P.rettgeri* P12105 chromosome | CP109846.1 | 99.65 | 100 | human |
| *Providencia* sp. PROV024 chromosome | CP120545.1 | 99.61 | 100 | pig |
| *P.rettgeri* W986 chromosome | CP076258.1 | 99.61 | 100 | unknown |
| *P.rettgeri* R39 chromosome | CP066315.1 | 99.61 | 100 | unknown |
| *Providencia* sp. PROV002 chromosome | CP096371.1 | 99.61 | 100 | human |
| *Providencia* sp. PROV003 chromosome | CP096369.1 | 99.61 | 100 | human |
| *P.rettgeri* YK205 chromosome | CP090217.1 | 99.61 | 100 | rearing water |
| *P.rettgeri* PROV002 chromosome | CP059345.1 | 99.61 | 100 | human |
| *P.rettgeri* HH18 chromosome | CP054158.1 | 99.37 | 100 | unknown |
