# Supplementary material for: Identification and characterization of a novel chromosome-encoded aminoglycoside O-nucleotidyltransferase gene, ant(9)-Id, in Providencia sp. TYF-12 isolated from the marine fish intestine
Source: Front Microbiol. 2024 Dec 12;15:1475172. doi: 10.3389/fmicb.2024.1475172 (PMC11669914; doi:10.3389/fmicb.2024.1475172)
Supplement: Supplementary file 12 [file Supplementary_file_1.docx]

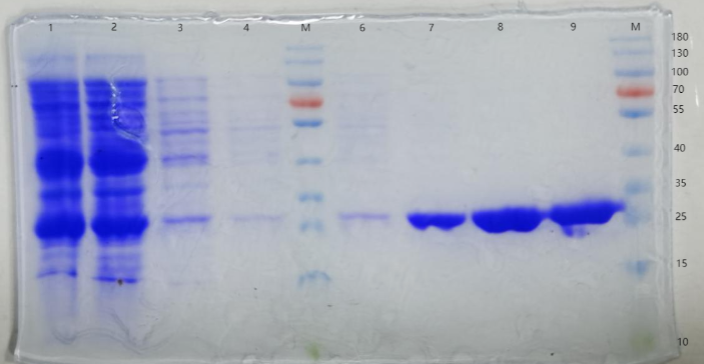


**FIGURE S1** | The SDS-PAGE analysis of the purified ANT(9)-Id protein. Count from left to right, lane 1, flow through; lane 2-4, wash 1-3; lane 5 (M) and 10 (M), markers; lane 6-9, elution 1-4.
